# Drag Reduction Using Polysaccharides in a Taylor–Couette Flow

**DOI:** 10.3390/polym9120683

**Published:** 2017-12-07

**Authors:** Pallavi Bhambri, Ravin Narain, Brian Fleck

**Affiliations:** 1 Department of Mechanical Engineering, University of Alberta, 9211-116 Street NW, Edmonton, AL T6G 1H9, Canada; bhambri@ualberta.ca; 2 Department of Chemical & Material Engineering, University of Alberta, 9211-116 Street NW, Edmonton, AL T6G 1H9, Canada

**Keywords:** drag reduction, polysaccharides, Taylor–Couette flow, turbulent flow

## Abstract

Three different polysaccharides, aloe vera, Tamarind powder and pineapple fibers, are utilized as drag reducing agents in a turbulent flow. Using a Taylor–Couette setup, consisting of a rotating inner cylinder, for measuring the drag reduction, a range of Reynolds numbers from 4 × 10^4^ to 3 × 10^5^ has been explored in this study. The results are in good agreement with previous studies on polysaccharides conducted in a pipe/channel flow and a maximum drag reduction of 35% has been observed. Further, novel additives such as cellulose nanocrystals (CNC), surfactants and CNC grafted with surfactants are also examined in this study for drag reduction. CNC due to its rigid rod structure reduced the drag by 30%. Surfactant, due to its unique micelle formation showed maximum drag reduction of 80% at low *Re*. Further, surfactant was grafted on CNC and was examined for drag reduction. However, drag reduction property of surfactant was observed to be significantly reduced after grafting on CNC. The effect of Reynolds number on drag reduction is studied for all the additives investigated in this study.

## 1. Introduction

The pressure drop in closed conduits is significant in turbulent flow and a considerable amount of energy is depleted in pumping the fluids to overcome this pressure drop, particularly in rough walled geometries. Reduction in this drag leads to substantial amount of cost and energy savings, so it is not surprising that it has been an area of extensive research in the past few decades. 

Drag can be either reduced by modifying the surface of pipe/channel or by adding some additives such as polymers, surfactants, fibers, bubbles, etc. Drag Reduction (DR) using high molecular weight polymers as additives is well documented and, after it was first discovered during the Second World War [1], an enormous amount of work has been carried out in this field. Additive concentration in the order of parts per million of these dissolved polymers has been shown to diminish the drag by 60–70% [2]. However, due to their high molecular weight (1–25 million Da), these materials have the disadvantage of being susceptible to mechanical degradation, i.e., chains get broken at higher strain rates [3]. The phenomenon of drag reduction has not been clearly understood despite several attempts being made by various researchers. Virk [4,5] and Lumley [6] have contributed significantly in understanding the DR mechanism. Virk [4,5] observed the onset of drag reduction and concluded that turbulence is required for drag reduction to occur. Lumley [7] provided a time criterion hypothesis to explain the mechanism of drag reduction and postulated that a coil stretch transition of the polymer chains is the reason behind DR. Polymer chains are significantly stretched in this transition state, which increases the elongational viscosity by ten thousand fold. This increase in elongational viscosity, which is prominent near the wall regions, dampens the turbulent structures thereby decreasing the drag. 

Surfactants such as hexadecyltrimethylammonium bromide (CTAB) have also been studied widely for reducing drag [8,9]. Micelle formation (microscopic units assembled from the molecules of surfactants) of the surfactants above the critical micelle concentration exhibit viscoelastic properties similar to polymers which remarkably diminishes the drag. Surfactants have an advantage over polymers due to their tendency to self-repair, i.e., if the micelle structure is broken by strain, they can automatically reorganize once the shear stress is lower, thereby restoring the drag reduction effect [10]. Similarly, fibers have also been reported as drag reducing additives. This mechanism is referred to as solid suspension drag reduction. Turbulence suppression by fibers weakens the transverse momentum transport, which subsequently reduces turbulent dissipation [11].

Currently, synthetic polymers such as polyacrylamides, polyethylene oxide, polyisobutylene, etc. are widely utilized as drag reducing agents in industry. However, due to environmental impact caused by slower degradation of these high molecular weight polymers, attention has been shifted to biopolymers. Plant polysaccharides such as xylan, guar gum, etc. have been identified as a natural substitute to synthetic Drag Reducing Agents (DRA) and these have the advantage of being biodegradable [12,13]. Singh and Rao [14] observed 70% drag reduction using guar gum at 500 PPM concentration, whereas Abdulbari et al. [15] used 400 PPM okra mucilage to achieve 60% DR. In addition, aloe vera has demonstrated 62% DR at *Re* of 10,000 in a pipe flow with 400 PPM concentration.

In the present study, three different polysaccharides: aloe vera; Tamarind powder and pineapple fibers are studied for DR. Aloe vera is composed of mucilaginous polysaccharides and contains different proportions of mannose, glucose and galactose. Pineapple fibers consist of a cellulose, hemicellulose and lignin; and possess good mechanical properties. Tamarind seed powder is a high molecular weight branched polysaccharide consisting of a cellulose-like backbone that carries xylose and galactoxylose substituents. 

Along with polysaccharides, pure cellulose nanocrystals (CNC) and surfactant grafted cellulose nanocrystals are also employed as DRA in this study. Cellulose nanocrystals are rod-like cellulose crystals, which possess hydroxyl groups and negative charge on the surface. Due to their favorable mechanical properties and high surface to volume ratio, these are widely studied as a composite material. Further, due to functional groups available on the surface, their surface can be easily modified [16]. Here, hexadecyltrimethylammonium (CTAB) bromide, a cationic surfactant, is used to graft to the surface of CNC and then further utilized for DR. CNC and surfactant grafted CNC has not been previously studied for DR.

Instead of commonly used pipe/channel flow, a Taylor–Couette setup is used for measuring the Drag reduction in this work as it has several advantages. It is a fluid motion between two coaxial cylinders with one or both the cylinders co-rotating/counter-rotating and possesses similar characteristics of a turbulent boundary layer [17]. The TC setup has been proven as a convenient and reliable testing platform for drag reduction using both additives and super hydrophobic coatings [18,19,20,21]. Specifically, it allows for good control of the fixed batch of fluid and detailed monitoring of DRA degradation as a function of time as well as energy dissipation. Torque measured on either inner or outer cylinder of this setup has been found to scale well using dimensionless torque (*G = T/*(*ρν*^2^*L*) [22], where *T* is the torque, *ρ* is the density of fluid, *ν* is the kinematic viscosity and *L* is the length of inner cylinder). Skin friction coefficient (*c_f_*) is then calculated from dimensionless torque by:

(1) $$c_{f}=\frac{G}{Re^{2}}$$

Drag reduction can be computed using Equation (2), where *c_f,w_* is the skin friction coefficient of water and *c_f,s_* is the skin friction coefficient solution with drag reducing additives.

(2) $$DR\%=\frac{c_{f,w}-c_{f,s}}{c_{f,w}}\;\times \;100$$

## 2. Materials and Methods

### 2.1. Experimental Design

**Figure 1:**
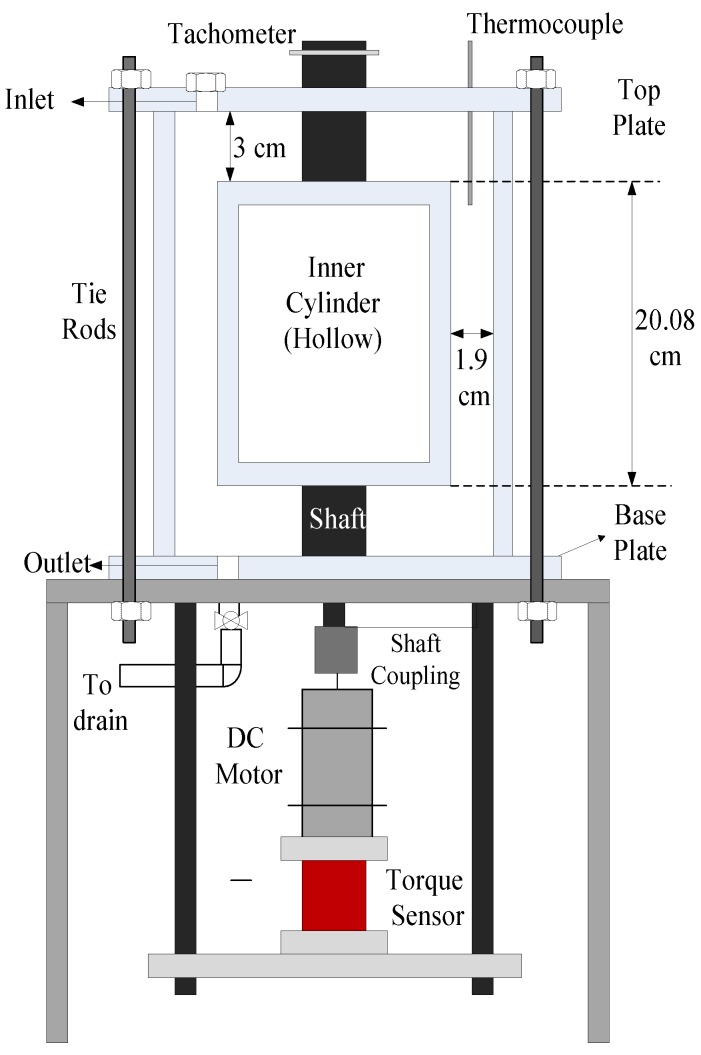
Schematics of Taylor–Couette Setup used in this study [18]. (Reproduced with permission from Wiley & Sons, Hoboken, NJ, USA).

The employed Taylor–Couette system has a rotating inner cylinder and a stationary outer cylinder, both of them manufactured using acrylic, and has been previously used [18,19] successfully (Figure 1). The system is closed at both the ends with static end plates. The radius of the inner cylinder (*r_i_*) is 6.03 cm whereas, the radius of the outer cylinder (*r_o_*) is 7.94 cm which provides a radius ratio (η = *r_i_*/*r_o_*) of 0.76. The inner cylinder has a length (*L*) of 20.08 cm, which leads to an aspect ratio [Г = *L*/(*r_o_* − *r_i_*)] of 10.56. A 120 V AC, NEMA 34, 1/3 hp speed control DC Motor (Amatek Inc., Berwyn, PA, USA) with a range from 300–3450 RPM, is connected to an anodized aluminum shaft. This shaft is fixed to the hollow inner cylinder using O-rings and shaft collar. Tie rods are used to connect the cylinders to end plates. A reaction torque sensor (TFF425, Futek Advanced Sensor Technology, Inc., Irvine, CA, USA), with a capacity of 7 N-m and 2 m V/V of rated output, is mounted at the base of motor which measures the reaction torque acting on the inner cylinder. The sensor was calibrated by Futek Advanced Sensor Technology, Inc. and had an error of 0.02% of rated output in the clockwise direction and −0.03% of rated output in the anti-clockwise direction. An optical tachometer was utilized to measure the angular velocity of the inner cylinder and a type K thermocouple was used to monitor the temperature of the fluid in the annular gap. The temperature difference before and after every reading was found to be less than 0.2 °C. The Reynolds number (*Re*) was calculated by Equation (3), where $\Omega_{i}$ is the angular velocity of the inner cylinder and ν is the fluid kinematic viscosity.

(3) $$Re=\;\frac{\Omega_{i}r_{i}\left(r_{o}-r_{i}\right)}{\nu }$$

### 2.2. Preparation of Polysaccharide Solution

Aloe vera, pineapple fibers and Tamarind powder, obtained from Chulalongkorn University, were used in the study. The same technique was used to prepare all the polysaccharide samples. A concentrated solution (2.4% *w*/*v*) was first prepared by sonicating the polysaccharides in water for 30 min, followed by stirring for 1 h. Solutions were then diluted and the final concentration of 600 PPM was utilized in the study for DR measurement.

### 2.3. Preparation of CNC, Surfactant and CNC-Surfactant Solution

Cellulose nano-crystals supplied by Alberta Innovates Tech Futures, with a particle length of 100–200 nm and a diameter 5–15 nm, were used. For pure CNC solution, a concentrated (2 wt %) solution was first prepared by sonicating CNC in water for 30 min followed by overnight stirring. Final concentration of 600 PPM was then utilized. 

For preparing the CNC-Surfactant (50/50) solution, the method provided by Kaboorani and Riedl [23] was used. Briefly, 2.4 wt % of CNC solution was obtained using 30 min of sonication, followed by 3 h of stirring. The 2.4 wt % CTAB (Sigma Aldrich, Oakville, ON, Canada) solution was prepared by magnetic stirring for 4 h. Both the solutions were then mixed and stirred for overnight. CNC has negative charge on the surface whereas CTAB is a cationic surfactant which leads to grafting of surfactant on CNC during mixing. Mixture was then centrifuged for 12 min at 15,000 RPM to remove the excess surfactants. CNC suspension was finally freeze dried for 2 days. A 600 PPM concentrated solution was prepared in water by magnetic stirring for 4 h. A 600 PPM solution of surfactant was also separately prepared and tested as a control.

### 2.4. Characterization of Additives

To calculate the *Re*, the viscosity of the solutions was measured using a rheometer (Anton Paar, RheolabQC, Montreal, QC, Canada) by varying shear rate from 1 to 1000 s^−1^ at 21 °C. The molecular weight of the aloe vera was determined by gel permeation chromatography (GPC) at room temperature and a Viscotek model 250 dual detector (refractometer/viscometer in aqueous eluents (0.5 M sodium acetate and 0.5 M acetic acid)) with a flow rate of 0.5 mL/min. Dynamic light scattering (DLS) was used to measure the effective diameter of pineapple fibers, Tamarind powder, CNC, Surfactant and CNC-Surfactant in the solution. Dynamic light scattering (Brookhaven Inc., Holtsville, NY, USA) was performed at a fixed angle y = 90° with an incident light of wavelength λ = 658 nm. Polysaccharide samples were also analyzed under scanning electron microscope (SEM, Philips/FEI XL30, Hillsboro, OR, USA). The Samples for SEM were coated with gold-palladium prior to imaging. Thermogravimetric analysis (TGA) was carried out using a SDTQ600 (TA Instruments, New Castle, DE, USA) analyzer between 10 °C and 700 °C in air at a heating rate of 10 °C min^−1^.

**Figure 2:**
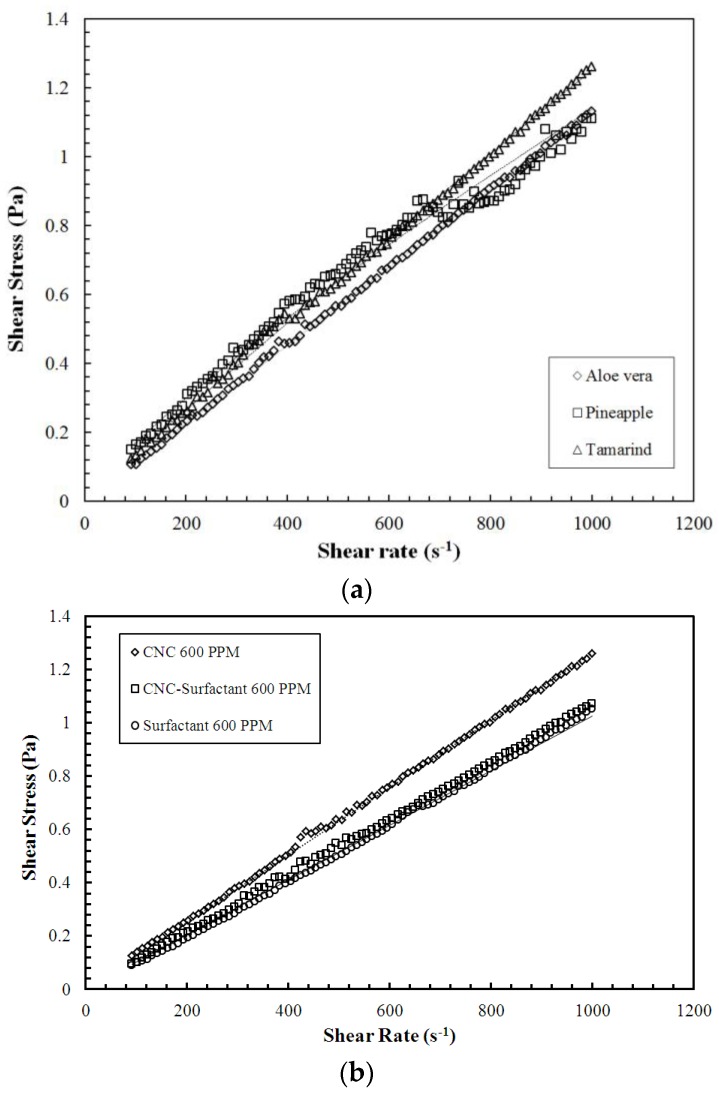
Shear Stress vs. Strain Rate for: (**a**) Polysaccharides; and (**b**) for CNC and CNC-Surfactant at 600 PPM. (The dotted line in (**a**,**b**) indicates the trendline using Power Law Fit).

**Figure 3:**
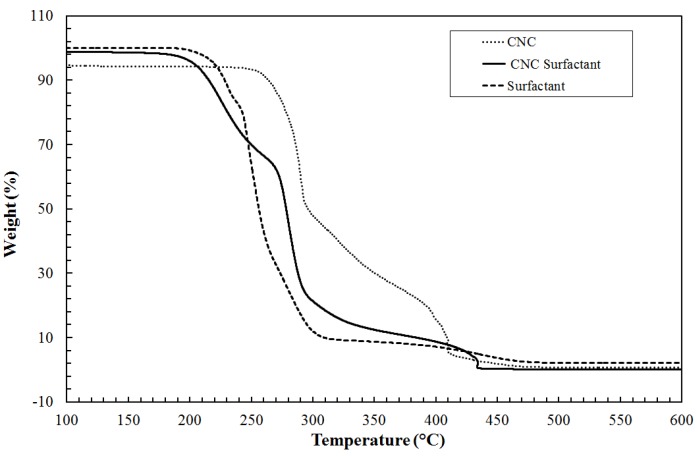
Thermogravimetric analysis of CNC and CNC Surfactant.

Figure 2 and Figure 3 shows shear stress (*τ*) vs. strain rate (*γ*) for polysaccharides and CNC, respectively, measured using rheometer. In the case of polysaccharides, aloe vera shows Newtonian behavior, whereas pineapple fibers and Tamarind powder demonstrate non-Newtonian behavior. Both CNC and CNC-Surfactant exhibited Newtonian behavior. Power law model $\tau \;=\;K{\frac{\partial u}{\partial y}}^{n}$ was used to fit the curves; where $\frac{\partial u}{\partial y}\;$ is strain rate (*γ*) and $K{\frac{\partial u}{\partial y}}^{n-1}\;$ represents viscosity (*μ*) (Table 1). 

Viscosity obtained from the above-mentioned expressions was used to calculate the *Re*. In the case of the non-Newtonian fluid, average of the viscosity between Shear Rate of 200 and 1000 s^−1^ was considered.

**Figure 4:**
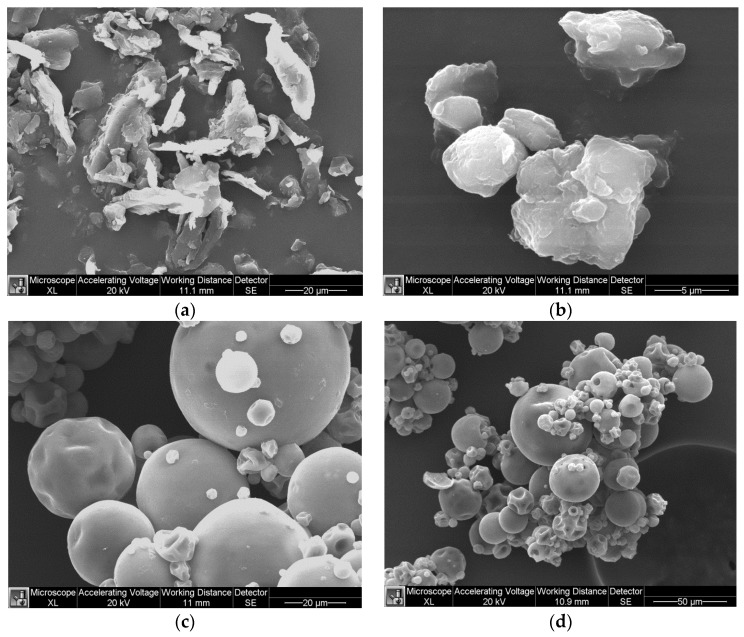
SEM images of: (**a**) Pineapple fibers; (**b**) Tamarind Powder; and (**c**,**d**) Aloe Vera.

The molecular weight (*M*_w_) of aloe vera was measured to be 280,000 Da indicating a sufficiently high molecular weight for drag reduction. Effective diameter of additives measured using DLS is shown in Table 2. The size of CNC-Surfactant is more than CNC alone, thereby confirming the grafting of CTAB on CNC. Figure 3 shows the TGA curves for CNC and CNC surfactant, demonstrating early decomposition of CNC Surfactant as compared to CNC. The surfactant begins to decompose at 230 °C whereas CNC will degrade at 300 °C. From these observations, the rough composition of CNC in CNC-Surfactant is estimated to be 55%. Figure 4 shows the SEM images of polysaccharides. Pineapple fibers consist of flakes and rigid rod type structure with approx. length of 20 µm and diameter of 1–2 µm with a huge variation in size. On the other hand, Tamarind powder consists of spherical particles with diameter of 2.5 µm. In the dried form, particles were agglomerated, as seen in the Figure 4b. Aloe Vera was used in a dissolved state; however, SEM images were taken to visualize the shape of Aloe Vera particles in the dried form. Spherical particles with huge polydispersity can be seen in Figure 4c,d.

## 3. Results and Discussion

### 3.1. Drag Reduction Using Polysaccharides

**Figure 5:**
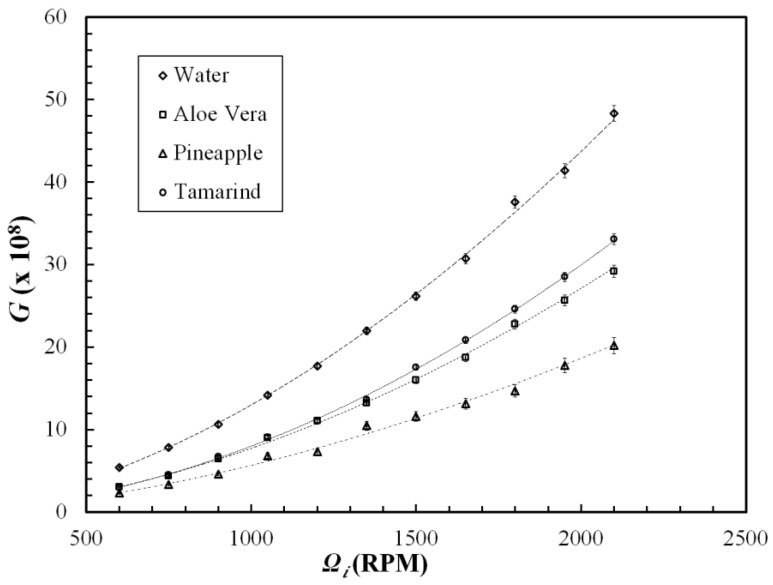
Dimensionless torque vs. angular velocity for polysaccharides.

Figure 5 shows dimensional torque (*G*) at various inner cylinder angular velocities (*Ω_i_*) for polysaccharides. Each point represents the average of three repeat measurements, while error bars indicates the data variability in the measurements. With increasing angular velocity, the difference between the dimensionless torque for the water and for the polysaccharide solutions is observed to increase with this phenomenon being most pronounced for pineapple fibers. This is in good agreement with the observations made by Campolo et al. [24] for xanthan gum in pipe flow.

**Figure 6:**
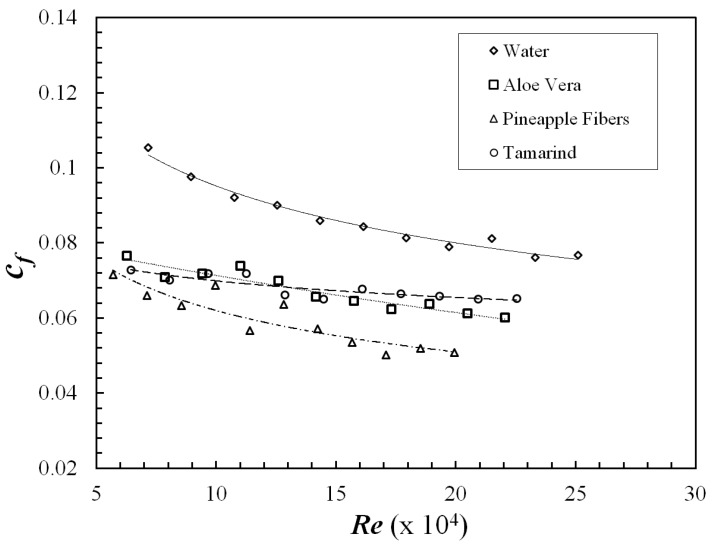
Skin friction coefficient vs. Reynolds number for polysaccharides.

**Figure 7:**
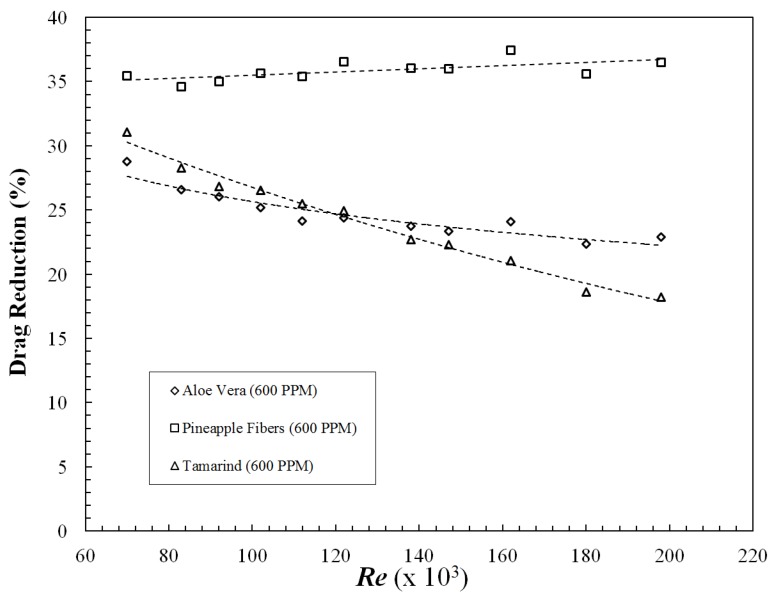
Drag reduction of polysaccharides with increasing Reynolds number.

Figure 6 indicates skin friction coefficient vs. *Re* for the polysaccharides and Figure 7 shows drag reduction (%), which is interpreted from skin friction coefficient (Equation (2)). Pineapple exhibited similar behavior to xanthan gum (Campolo et al., 2015), and a slight increase in DR% with increasing *Re* can be seen. On the contrary, aloe vera and Tamarind showed decreasing DR% with increasing *Re*. This is in agreement with the observations made by Abdul Bari et al. [15,25] for aloe vera and okra mucilage, respectively. On the other hand, synthetic polymers such as polyacrylamide and polyethylene oxide exhibit increased DR up to a critical *Re* and then a constant DR% beyond this critical *Re* [4,10,26]. This could be attributed to poor shear stability of polysaccharides in comparison to synthetic polymers. Contrarily, Fibers have good stability at high shear stress [27], which can also be observed in the case of Pineapple fibers in the currents study. It can be concluded that, among the polysaccharides considered in this study, Pineapple fibers and Tamarind had the best and worst resistance to shear degradation respectively. A comparison was also conducted at lower concentration of 200 and 400 PPM. Tamarind showed similar DR at both the lower concentration; however, Aloe Vera and Pineapple demonstrated negligible DR at these concentrations. Hence, to make an appropriate comparison, 600 PPM concentration was utilized. 

### 3.2. Drag Reduction Using CNC, Surfactant and CNC-Surfactant

**Figure 8:**
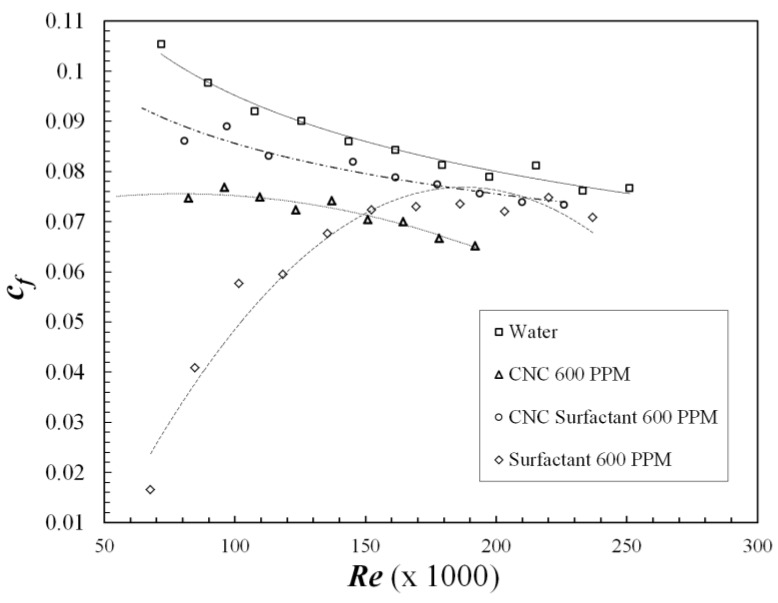
Skin friction Coefficient vs. Reynolds number for CNC, Surfactant and CNC-Surfactant.

**Figure 9:**
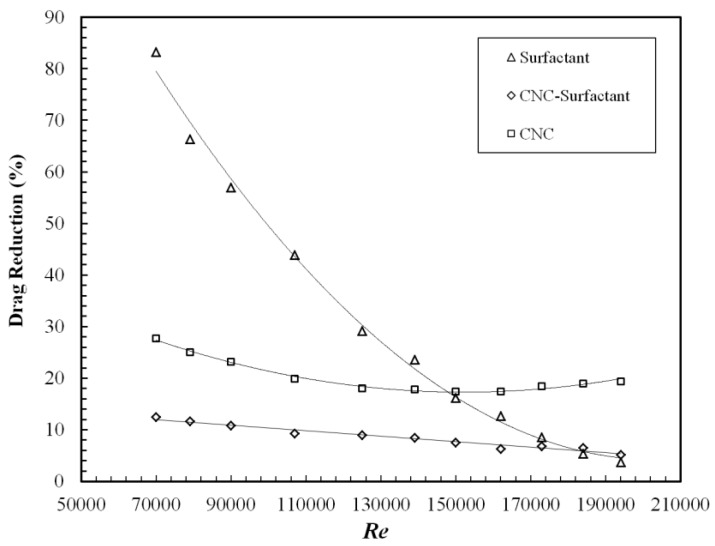
Drag reduction with increasing Reynolds number for CNC, Surfactant and CNC-Surfactant.

Figure 8 and Figure 9 show skin friction coefficient and drag reduction, respectively, for Surfactant, CNC and CNC-Surfactant. Drag reduction with surfactants can be observed to sharply decrease with increasing *Re*. The formation of micelle structures is the reason behind the DR in case of surfactants, however these thread-like structures easily break up at higher shear stress or *Re*, making them ineffective at large *Re*. These results agree well with the observations made by Zhang et al. [28]. On the other hand, due to the rigid rod structure of CNC, it exhibits only 30% DR which varies slightly with the *Re*. Due to their non-deformable structure, the size and shape of CNC does not vary much with strain rate which leads to nearly constant DR with increasing DR. This is consistent with the drag reduction shown by colloidal crystals of milling yellow dye having an aspect ratio of 5.7. However, grafting of surfactant on CNC reduced the DR to 10%, indicating the obtained structure does not exhibit viscoelastic properties similar to micelles in the case of pure surfactants. It could be either due to poor grafting density of surfactants on CNC or the rigid structure of CNC, which is at the core of surfactants in this structure. Due to weak bonding between CNC and surfactant, DR can be observed to decrease with increasing *Re*. Similar to polysaccharides, DR for these samples were also tested at lower concentrations of 200 PPM and 400 PPM. CNC-Surfactant and CNC demonstrated similar DR at these lower concentrations; however, the surfactant showed negligible DR at lower concentrations. The surfactant required a critical concentration beyond which the micellar structure is formed, which possesses the viscoelastic properties required to demonstrate DR. Since 600 PPM concentration was observed to the appropriate concentration for surfactant, the same concentration was chosen for CNC and CNC Surfactant for comparison. 

## 4. Conclusions

Additives such as aloe vera, pineapple fibers and Tamarind powder are explored as drag reducing agents using a Taylor–Couette flow setup. These polysaccharides exhibit 35% of DR at fairly low concentration of 600 PPM and have an advantage of being biodegradable as compared to Synthetic polymers. However, these have the drawback of being more susceptible to mechanical degradation and show decreased DR with an increase in *Re*. The current study also investigates cellulose nanocrystals, both rigid rod and biodegradable cellulose crystals with aspect ratio varying from 10 to 20, for DR. Due to their non-deformable structure, CNC demonstrated roughly constant DR with an increase in strain rate or *Re*. Further, a cationic surfactant (CTAB) was grafted to the CNC and was then subsequently investigated as drag reducing additives. The surfactant was also investigated separately for DR to make a comparison and showed 80% DR at low *Re* which significantly dropped down with increase in *Re*. However, CNC-surfactant exhibited reduced DR as compared to CNC and surfactant separately, signifying that the grafted structure does not show similar viscoelastic properties as the micelle formation of the surfactant. In summary, CNC and other polysaccharides studied in this paper are identified as a potential biodegradable and environmentally friendly drag reducing additives. 

## Figures and Tables

**Table 1:** Power law model expression for different polysaccharides.

Polysaccharides	Power Law Expression
Aloe Vera	*τ* = 0.0011 *γ*
Tamarind Powder	*τ* = 0.0016 *γ*^0.9649^
Pineapple fiber	*τ* = 0.0037 *γ*^0.8307^
CNC	*τ* = 0.0013 *γ*
CNC-Surfactant	*τ* = 0.0011 *γ*
Surfactant	*τ* = 0.0010 *γ*

**Table 2:** Size of the additives measured using Dynamic light scattering (DLS).

Additive	Effective Diameter (nm)	Standard Deviation (nm)
**Tamarind**	2466	414
**CNC**	736	24
**CNC-Surfactant**	1877	300
**Surfactant**	214	45
